# Tolerogenic vaccines for the treatment of cardiovascular diseases

**DOI:** 10.1016/j.ebiom.2020.102827

**Published:** 2020-06-20

**Authors:** Fernando Lozano Vigario, Johan Kuiper, Bram Slütter

**Affiliations:** Division of BioTherapeutics, Leiden Academic Centre for Drug Research, Einsteinweg 55, PO Box 9502, 2300RA Leiden, the Netherlands

**Keywords:** Atherosclerosis, Inflammation, Tolerance, Vaccination

## Abstract

Atherosclerosis is the main pathology behind most cardiovascular diseases. It is a chronic inflammatory disease characterized by the formation of lipid-rich plaques in arteries. Atherosclerotic plaques are initiated by the deposition of cholesterol-rich LDL particles in the arterial walls leading to the activation of innate and adaptive immune responses. Current treatments focus on the reduction of LDL blood levels using statins, however the critical components of inflammation and autoimmunity have been mostly ignored as therapeutic targets. The restoration of immune tolerance towards atherosclerosis-relevant antigens can arrest lesion development as shown in pre-clinical models. In this review, we evaluate the clinical development of similar strategies for the treatment of inflammatory and autoimmune diseases like rheumatoid arthritis, type 1 diabetes or multiple sclerosis and analyse the potential of tolerogenic vaccines for atherosclerosis and the challenges that need to be overcome to bring this therapy to patients.

## 1 Inflammation and autoimmunity in atherosclerosis

Atherosclerosis is the main underlying cause of acute cardiovascular events such as myocardial infarction or stroke [1]. Traditionally, atherosclerosis has been primarily considered a pathology related to dyslipidaemia, therefore lipid-lowering therapies such as statins have been the mainstream option for prevention of cardiovascular events. However, in recent years several lines of evidence have highlighted the importance of the immune component of atherosclerosis [2]. The development of an atherosclerotic lesion initiates with the infiltration of lipoproteins, mainly low-density lipoproteins (LDL), from the blood into the intima of the arteries. The infiltration and accumulation of LDL particles into the arterial intima leads to the chemical modification of some components of the lipoprotein and the formation of modified LDL, mainly oxidized LDL (oxLDL). The presence of oxLDL in the sub-endothelial space of the artery triggers an inflammatory response that involves both the innate and the adaptive arms of the immune system [3].

The initial accumulation of oxLDL leads to the over-expression of adhesion molecules in endothelial cells, promoting the infiltration of monocytes from the blood. These monocytes will differentiate into macrophages, which phagocyte LDL particles and eventually become foam cells, characterized by the presence of large cholesteryl esters deposits in the cytoplasm and the secretion of pro-inflammatory cytokines like IL-1β [4].

Other innate immune cells such as dendritic cells (DCs) play an important role in the development of the plaque. DCs, a subset of antigen-presenting cells, are the link between the innate and the adaptive immune responses. DCs in the atherosclerotic plaque phagocyte antigens such us LDL from the environment, process them and present small apoB100-derived peptides bound to Major Histocompatibility Complex (MHC) molecules in their surface. These cells mostly migrate to draining lymph nodes, present ApoB100 peptides to naïve T cells and stimulate their differentiation and expansion into antigen-specific effector T cells. In the plaque, these effector T cells will recognize the antigens presented by antigen-presenting cells, releasing pro-inflammatory cytokines such as IFN-γ that contribute to the development of the lesion [5,6].

There are several subsets of effector T cells with a role in atherosclerosis. T helper (Th) CD4^+^ cells form a major population in the plaque next to CD8^+^ *T* cells [7]. The different subsets of Th cells might have completely different roles in the development of atherosclerosis. For instance, Th1 cells have a pro-atherogenic effect due to the secretion of pro-inflammatory cytokines, mainly IFN-γ [8]. On the other hand, T regulatory cells (Tregs) are a subset of Th cells that are thought to have anti-atherogenic functions as lower frequency and functional impairment of Tregs have been observed in patients with coronary artery disease [9,10]. Tregs can suppress effector T cells, such as Th1 cells, and therefore control inflammatory responses.

The important role of LDL in the initiation of atherosclerotic lesions, the presence of both auto-reactive T and B cells as well as anti-oxLDL antibodies has led to the notion of the autoimmune component of atherosclerosis [11,12]. Interestingly, there is a well-defined correlation between autoimmune disorders and the development of atherosclerosis [13]. For instance, rheumatoid arthritis patients have a higher risk of cardiovascular disease and have a 1.5 to 2-times higher rate of cardiovascular events than the general population [14,15].

The importance of the immune component of atherosclerosis is evidenced by the presence of residual cardiovascular risk in a significant percentage of patients undergoing lipid-lowering therapies, highlighting the need of novel approaches in the treatment of cardiovascular diseases (CVD) [16].

## 2 Anti-inflammatory therapies in clinical trials

Despite the widespread use of statins, cardiovascular diseases are still the number 1 cause of death globally. This can be partially explained by the presence of the significant residual cardiovascular risk observed in patients even under intensive statin treatment and low levels of LDL [17]. Randomized clinical trials have shown that the frequency of cardiovascular events is lower in patients with low levels of high-sensitivity C-reactive protein (hsCRP), a biomarker of inflammation [18]. These observations suggest that targeting inflammation in atherosclerosis might contribute further to the reduction in cardiovascular risk [19].

Targeting pro-inflammatory cytokines to reduce the residual inflammatory risk of cardiovascular events is a strategy that has been studied in clinical trials. The CANTOS (Canakinumab Antiinflammatory Thrombosis Outcome Study) trial assessed the efficacy of the anti-IL1β human monoclonal antibody canakinumab to prevent vascular events in patients with previous history of myocardial infarction and presenting high levels of hsCRP marker. IL-1β, a pro-inflammatory cytokine, triggers the upregulation of inflammatory markers and adhesion molecules in endothelial cells, promotes the recruitment of immune cells to the atherosclerotic plaque and induces smooth muscle cell proliferation [20,21]. Altogether, this contributes to the initiation and development of atherosclerosis plaques [22]. Patients included in the CANTOS trial had undergone aggressive secondary prevention therapies including high-dose of statins. The results showed that the treatment with 150 mg of Canakinumab once every 3 months leads to a 15% reduction in the incidence of non-fatal myocardial infarction, non-fatal stroke or cardiovascular death compared to placebo. The results also showed a significant reduction in the inflammatory biomarkers hsCRP and IL-6 but no effects on LDL or HDL levels, therefore the clinical benefit could be attributed to the attenuated inflammation [23]. The results from this study point out to the importance of targeting the cardiovascular risk associated to inflammation in addition to the reduction of LDL cholesterol levels.

Similarly, results from COLCOT (Colchicine Cardiovascular Outcomes Trial) have shown that low-doses of colchicine (0.5 mg/day), a potent anti-inflammatory drug, led to a significant reduction in the risk of death from cardiovascular causes, resuscitated cardiac arrest, myocardial infarction, stroke and angina compared to the placebo group [24].

Another approach to target inflammation in atherothrombosis is the use of low doses of methotrexate. Low-dose methotrexate is already used in the treatment of certain inflammatory conditions and observational studies have associated this treatment with lower frequency of cardiovascular events [25]. However, in the Cardiovascular Inflammation Reduction Trial (CIRT), low-dose methotrexate did not reduced the levels of inflammatory biomarkers IL-1β, IL-6 or CRP in patients with previous history of myocardial infarction or coronary disease and did not reduced the frequency of nonfatal myocardial infarction, nonfatal stroke, cardiovascular death or unstable angina [26].

The apparent contradictory results from CANTOS, COLCOT and CIRT studies highlight the complexity of the inflammatory pathways involved and the importance of taking this into consideration when designing anti-inflammatory interventions for cardiovascular diseases.

## 3 Antigen-specific tolerance in inflammatory and autoimmune diseases

The presented therapeutic strategies induce a systemic anti-inflammatory state that can have important side effects in the long term, such as higher risk of infections, as shown in both the CANTOS and the COLCOT trials [27,23,24]. An alternative approach to the use of systemic anti-inflammatory molecules could be the induction of antigen-specific tolerance towards disease-specific autoantigens. This approach has been translated into clinical trials for diseases such as rheumatoid arthritis (RA), type 1 diabetes (T1D) and multiple sclerosis (MS), but has not been clinically applied for treatment of CVD. Lessons learned from these clinical trials can help to advance the application of this strategy in CVD.

Two main approaches for the induction of antigen-specific immune tolerance have been studied so far in clinical trials. One is the administration of tolerogenic dendritic cells (tolDCs) or Tregs with capacity to induce immune tolerance. Another approach is the administration of autoantigens at low doses, together with tolerogenic adjuvants or using tolerogenic administration routes.

An example of the former is a phase I clinical trial that studied the safety of the administration of tolDCs loaded with autoantigens from synovial fluid for treatment of RA [28]. For this trial, tolDCs were differentiated from peripheral blood mononuclear cells (PBMCs) from patients in the presence of dexamethasone and vitamin D3 [29]. Safety assessment was made based on the proportion of patients experiencing flares in the target knee within 5 days after administration of tolDCs. None of the patients reported aggravated symptoms indicating that the therapy is safe. Furthermore, an exploratory efficacy assessment showed symptoms resolution in 2 out of 3 patients receiving the highest cell dose.

In another phase I clinical trial, the safety and biological activity of autologous tolDCs loaded with the citrullinated peptides relevant for RA were administered intradermally to RA patients [30]. The safety of the treatment was confirmed as only mild adverse effects were detected. Furthermore, the results showed a decrease in the levels of CRP and pro-inflammatory cytokines and a reduction in the population of effector T cells. Unfortunately, clinical efficacy was not evaluated in this trial, mainly due to the low number of patients included in the trial. This limitation was related to the high costs associated to the manufacture of autologous tolDCs, one of the main drawbacks of cell-based therapies.

The administration of autologous tolDC also appeared to be safe and well-tolerated in a phase I clinical trial in T1D [31]. Safety was evaluated not only based on the presence of adverse reactions, but also by measuring the presence in serum of auto-antibodies and cytokines different to those usually present in T1D and following changes in immune cell populations with flow cytometry. Patients received intradermal injection of 1 × 10^7^ cells either not manipulated or differentiated into tolDCs. The results from the trial did not show any noticeable adverse effect on patients, neither significant changes in immune cell populations.

Another cell-based strategy to induce tolerance is the administration of Tregs. In a phase I clinical trial for T1D, the administration of autologous polyclonal Tregs showed that the therapy was well tolerated, and no major adverse reactions were observed [32]. Clinical parameters like c-peptide levels, insulin use and haemoglobin A1c levels were included as secondary outcomes in the study. However, due to the reduced number of patients enroled in the study (16 patients in 4 dose cohorts), no assessment of the clinical efficacy of the treatment could be done. Previous reports have highlighted potential problems with Treg cell therapies regarding phenotypic changes in the Tregs, switching from an anti-inflammatory to a pro-inflammatory phenotype or the contamination with effector T cells that could aggravate the disease [33,34]. Therefore, in this study, the stability of the Treg phenotype after *in vitro* expansion and the suppressive activity of the Tregs before the administration had to be closely monitored.

The other approach for the induction of antigen-specific tolerance is the use of peptides. A clinical trial in RA have studied the efficacy of tolerance induction towards heat-shock proteins (HSP). HSP are naturally produced by cells under certain stress situations such as inflammation. The release of HSP by stressed cells induces an anti-inflammatory response mediated by HSP-specific Tregs in order to control the inflammatory process [35]. In a phase II trial, RA patients received an oral dose of the HSP40-derived peptide dnaJP1 to induce mucosal tolerance. The peptide dnaJP1 has been previously identified as an important T cell epitope in RA [36]. The results showed a significant improvement in clinical response, defined by a reduction in swollen joints, pain, disability and/or acute phase reactant levels, in the group treated with dnaJP1 compared to placebo. Furthermore, this improvement was accompanied by a reduced production of pro-inflammatory TNFα in *ex vivo* re-stimulated PBMCs [37].

Results of clinical trials for peptide-based treatments in T1D are also promising. In a phase Ib trial, the intradermal administration of a proinsulin peptide to T1D patients showed to be safe and well tolerated [38]. The results showed a less pronounced loss of insulin production capacity in the treatment groups compared to the placebo group. Consequently, no significant increase in average insulin doses was observed in the treatment groups while a progressive increase was seen in the placebo group. Furthermore, treated patients showed higher levels of anti-inflammatory IL-10 production when CD4^+^ *T* cells were *ex vivo* stimulated with proinsulin.

In the context of MS, the application of tolerogenic vaccines has provided mixed results regarding their efficacy and tolerability. A phase II clinical trial in 2005 showed that the intravenous administration of the synthetic peptide MBP8298, derived from myelin basic protein (MBP), had a beneficial effect only in a subgroup of MS patients presenting HLA haplotypes DR2 and/or DR4. Patients receiving MBP8298 also showed a reduction in the level of anti-MBP autoantibodies, indicating tolerization towards the antigen [39]. However, in a phase III trial, the same peptide and dosage failed to show a significant reduction in disease progression in HLA-DR2^+^ and/or HLA-DR4^+^ patients [40].

Cocktails of different peptides derived from MBP have performed better in clinical trials. In phase Ib and IIa clinical trials, the administration of a cocktail composed of 4 peptides derived from MBP led to a reduced number of brain lesions compared to baseline. The trial design included a dose escalation period of 8 weeks, with peptide doses increasing from 25 µg to 800 µg followed by a full-dose (800 µg peptide cocktail) period in order to avoid unwanted T cell activation and systemic pro-inflammatory cytokine release, a common concern in these peptide-based therapies [41].

Other clinical trials have highlighted the importance of the administration route for the induction of tolerance. Oral, sublingual, nasal and dermal administration routes are the most commonly used due to the natural role of mucosal and skin immunity in tolerance induction [42]. For instance, the transdermal administration route has shown to be optimal to induce tolerance towards myelin peptides in MS patients. A clinical trial studied the administration of a mixture of three peptides derived from MBP, proteolipid protein (PLP) and myelin oligodendrocyte glycoprotein (MOG), to MS patients using a transdermal skin patch. Skin biopsies showed an increase in activated Langerhans cells, a type of antigen-presenting cells found in the skin, and an increase in DCs in draining lymph nodes in patients receiving the peptides mixture compared to the placebo group. The treatment also attenuated the proliferative response of CD4^+^ *T* cells, an increased production of IL-10 and a decrease in IFN-γ production upon *ex vivo* re-stimulation with the peptides. The study however did not include any clinical endpoints to assess the effect of the treatment in the progression of the disease [43].

Overall, the clinical trials have shown that tolerance induction is a feasible approach that can attenuate some of the pro-inflammatory responses present in diseases such as RA, T1D or MS but they have also shown the limitations of both peptide-based and cell-based therapies (Table 1). Peptide-based approaches present advantages such as relatively straight-forward development of stable formulations of peptides thanks to the extensive work carried out in the formulation of subunit vaccines for infectious disease as well as the lower cost of manufacture compared to cell-based therapies and often admit more convenient administration routes such as oral or intradermal. But this strategy also presents some disadvantages, for instance the dose should be carefully selected as too high doses can induce unwanted pro-inflammatory responses while too low doses will not have the desired tolerogenic effect. Furthermore, the proteolytic degradation of the peptide in biological fluids should also be taken into account when choosing the peptide dose. Lastly, appropriate HLA-peptide interaction is a prerequisite for the effectivity of peptide-based immunotherapies therefore HLA typing of patients is often required in this type of therapies, as described in the clinical trial with MBP8298 peptide [39]. The main advantage of cell-based therapies is that the administration of tolDCs or Tregs can directly induce immune tolerance through expansion of antigen-specific Tregs or induction of anergy in effector T cells, respectively. However, these therapies often involve the isolation of autologous cells from patients, the induction of a tolerogenic phenotype *ex vivo* and the subsequent reinfusion to the patient, which is costly and time-consuming [44]. Furthermore, there are concerns over the variability and stability of the tolerogenic phenotype induced *ex vivo*.

**Table 1:** Main advantages and disadvantages of peptide-based and cell-based strategies for the induction of tolerance.

Peptide-based therapies		Cell-based therapies	
Advantages	Disadvantages	Advantages	Disadvantages
Lower cost of manufacture	HLA genotyping often required	Direct tolerance induction	Expensive and time-consuming manufacture
Stable peptide formulations are relatively straight-forward	Peptides subject to proteolytic degradation		Variability of tolerogenic phenotype
Easier administration	Dose need to be carefully selected		

The formulation of peptide-based vaccines is crucial not only for stability of the peptide but also for the induction of tolerance. The use nanoparticles as delivery systems for immune modulation is currently under investigation and has the potential to overcome some of the problems associated with peptide and cell-based therapies. For instance, the delivery of peptides using nanoparticles can prevent degradation by proteases in biological fluids, allow the specific delivery to target cells such as DCs, include tolerogenic adjuvants to induce the desired tolerogenic phenotype *in vivo* and these nanoparticle formulations can be manufacture in high volumes at lower cost than cell-based therapeutics.

## 4 Tolerogenic vaccines in atherosclerosis

Despite the strong evidence supporting the role of inflammation and autoimmunity in the development of atherosclerosis, to the best of our knowledge no clinical trials have been carried out to study the safety, tolerability, feasibility or efficacy of antigen-specific tolerance induction to halt the development of atherosclerosis.

One of the obstacles in the development of vaccines for atherosclerosis is the lack of well-defined epitopes responsible for the initiation and maintenance of the inflammatory responses in atherosclerosis. However, several reports have shown that the induction of tolerance towards oxLDL and HSP attenuate atherosclerosis in pre-clinical models [45], [46], [47]. Therefore peptides derived from ApoB100 or HSP have been the main focus of attention in the search of relevant epitopes in atherosclerosis.

Oral administration of oxLDL or malondialdehyde-treated LDL induced Treg-mediated tolerance in LDLr^−/−^ mice, accompanied by a significant reduction in atherosclerotic lesions initiation and progression [46]. This report indicates the potential of ApoB100-derived antigens as candidates for peptide-based vaccination strategies in atherosclerosis. Immunization of LDLr^−/−^ (LDLr^tm1Her)^ mice expressing human ApoB100 with P210 (human ApoB100_3136-3155_) has shown to reduce atherosclerosis in pre-clinical studies and the mechanism of action of this therapeutic strategy has been related to the induction of Tregs. The administration of peptide P45 (human ApoB100_661-680_) or P210 together with alum salts adjuvant has shown to reduce the area of atherosclerotic plaques by 66 and 55% respectively compared to control. The authors also speculate with the activation of Tregs as the mechanism of action mediating this anti-atherogenic effect [48,49]. In another study, the administration of P210 without any adjuvant led to a 30% reduction in plaque size compared to control group and abrogated the growth of advanced atherosclerotic lesions. Furthermore, this effect was associated with a 30% increase in the Treg frequency in lymph nodes and Treg depletion abolished the atheroprotective effect of the immunization [50].

The immunization with peptide P6 (ApoB100_978-992_) together with Complete Freund's adjuvant (CFA) has shown to promote atherosclerosis in murine models while the same peptide can have atheroprotective effects when administered with a different immunization regime consisting in a prime immunization with P6 in CFA followed by 4 booster immunization with Incomplete Freund's Adjuvant (IFA), known to induce suppressive immune responses [51], [52], [53]. The same P6 peptide in combination with the squalene oil-based adjuvant Addavax also induced a 52% reduction in aortic lesion area in ApoE-^/−^ mice compared to peptide alone [54]. These results highlight the importance of the careful selection of adjuvants to achieve atheroprotection.

The selection of atheroprotective peptides to be used in a tolerogenic vaccine should also consider the binding affinity to different HLA haplotypes. Peptides that can bind to a wide range of HLA haplotypes, such as peptides P265 and P295 from human ApoB100 described by Gisterå et al. would cover most of the world population [55]. Similarly, peptide P18 (human ApoB100_3030-3044_) was identified *in silico* and *in vitro* as a strong binder to the most common HLA-DR haplotypes. Immunization of ApoE^−/−^ mice with P18 showed an induction of Tregs and a 35% reduction in atherosclerosis lesion size [56]. These studies underline the importance of taking into account the wide variability of HLA haplotypes from early phases of pre-clinical research.

The use of nanoparticles with intrinsic tolerogenic capacity, such as specific liposomal formulations, to deliver ApoB100 peptides has also shown promising results in murine models of atherosclerosis. The use of a liposomal formulation containing 1,2-distearoyl-sn‑glycero-3-phosphoglycerol (DSPG) to deliver the peptide P3500 (ApoB100_3500-3514_), similar to P3 peptide (ApoB100_3501-3515_) previously reported to induce atheroprotection by Ley group, showed to reduce lesion size by 50% compared to control [57,52].

Other antigen candidates for immunomodulatory vaccines for atherosclerosis are HSP-derived peptides. These proteins are over-expressed under cellular stress and they are involved in several inflammatory and autoimmune diseases including atherosclerosis [58,59]. HSP-specific Tregs could halt inflammation in atherosclerosis in a by-stander manner due to the constitutive over-expression of HSP in inflamed tissue. Therefore, the induction of HSP-specific Tregs could circumvent the problem of the lack of well-defined primary antigenic trigger in atherosclerosis [35]. Vaccination studies with HSP have provided mixed results, with some showing an increase in atherosclerosis upon immunization while others showed an anti-atherogenic effect [60], [61], [62], [63], [64]. A key difference between these studies might be the adjuvant used. In the studies showing a pro-atherogenic effect of HSP immunization, the protein was administered together with CFA or IFA. Contrarily, the studies showing an anti-atherogenic effect of the immunization with HSP proteins or peptides either use tolerogenic adjuvants, such as the combination of alum salts and anti-CD45RB antibodies, or are administered without adjuvants, and the effects have been linked to the induction of Tregs. Therefore, the election of the appropriate adjuvants and administration route to promote tolerance seems to be critical.

Overall, pre-clinical data supports effectiveness of tolerance induction towards atherosclerosis-relevant peptides to halt the development of atherosclerosis. The delivery of these antigens in the absence of TLR stimulation or in the presence of molecules that promote tolerance, can induce the expansion of Tregs and reduce the pro-inflammatory responses in atherosclerosis [50,54].

Most of the studies have focused on arresting the development of growing atherosclerotic plaques, however the majority of clinical manifestations of atherosclerosis occur at advanced stages of the disease. Therefore, the induction of atherosclerosis lesion regression and/or plaque stabilization is clinically most relevant. The potential of Tregs to induce plaque regression and stabilization has been shown in pre-clinical animal models. It has been reported that the administration of CD3 antibodies combined with a reduction in non-HDL cholesterol levels can increase the Treg to effector T cells ratio and induce 25% regression in plaque size in a mouse model of atherosclerosis. Furthermore, this effect was abolished upon Treg depletion, indicating a critical role of Tregs in the atherosclerosis regression [65]. In another study, Tregs were induced in a LDLr^−/−^ mouse model by administration of a IL-2 and anti-IL-2 antibody complex, achieving a 10-fold increase in Tregs. This Treg expansion led to stabilization of advanced atherosclerotic lesions established before starting the treatment [66]. These results show the potential of tolerogenic vaccines inducing Tregs expansion to stabilize and prompt regression of atherosclerotic lesions.

## 5 Clinical translation

Despite considerable pre-clinical research carried out in the field of tolerogenic vaccination for atherosclerosis, the promise of an atherosclerosis vaccine remains unfulfilled. There are several challenges in the translation from pre-clinical models to clinical trials. First of all, the animal models to study atherosclerosis present important difference with the human situation. For instance, ApoB expressed in mice undergoes different post-translational modifications and has a different structural conformation than human-expressed ApoB, and therefore can trigger different immune responses [67]. Furthermore, the immune system of mice presents considerable differences with human, such as several cytokines and immune receptors [68]. The use of humanized mice that resemble more closely the human situation, for instance expressing human ApoB100 or with humanized immune system, can overcome some of these challenges [48,69].

A prerequisite for the clinical translation of this therapeutic strategy is the selection of antigen or antigens for vaccination. It is not clear whether there is a common driving antigen for all HLA haplotypes in atherosclerotic patients. Furthermore, specific subtypes of HLA, such as HLA-DRB1×01, have been associated with increased risk of coronary artery disease and myocardial infarction [70]. This can be important in the selection of antigens for potential vaccine as it might be interesting to select peptides with strong binding affinity for those HLA haplotypes more prevalent in atherosclerosis patients. Therefore, it is yet to be determined if a broadly applicable vaccine can be developed or a personalized strategy should be followed instead.

The selection of the vaccine formulation is another important point to carefully consider. Initially, the most obvious formulations would include the peptide or peptides and the appropriate adjuvants, resembling formulations of subunit vaccines that have been widely investigated. Further developments should include nanoparticle formulations such as delivery systems with intrinsic tolerogenic capacity [57].

The next step is the design of clinical trials for a tolerogenic vaccine strategy and this will require a careful selection of the target population. Should only patients with high inflammatory risk be admitted or should any patient with clinically confirmed atherosclerosis be eligible? For this decision we should consider what design will better show the potential for this therapeutic approach. For instance, patients presenting high inflammatory risk would potentially benefit the most from the therapy and perhaps should be the focus of the first trials.

Finally, another parameter that can be challenging is the selection of the endpoints of the clinical trials. This requires the definition of biomarkers for atherosclerotic progression, regression and cardiovascular risk. The assessment of the atherosclerotic plaque morphology, size or parameters related to plaque stability could be done using imaging techniques such as tomography, however this would represent a significant technical challenge in large clinical studies. Due to the immune-modulating nature of the therapeutic approach, the monitoring of both humoral and cellular adaptive immune responses, as well as the antigen-specificity of those responses would be necessary. Furthermore, the levels of inflammatory biomarkers like CRP or pro-inflammatory cytokines like IL-6 or IL-1β, have shown to be good predictors of cardiovascular risk and should be used to monitor the efficacy of an atherosclerosis vaccine [71].

## 6 Conclusions

Cardiovascular diseases and their main underlying pathology, atherosclerosis, remain amongst the top causes of mortality globally. High levels of LDL cholesterol were once considered the only driving force underlying the formation and progression of atherosclerotic plaques. However, despite the broad use of lipid-lowering therapies, a significant proportion of patients remains at risk for cardiovascular complications. Recent evidence shows the importance of inflammation and a possible autoimmune component in the aetiology of atherosclerosis, but no therapies have been developed yet to target this key component of the disease. The induction of immune tolerance towards the self-antigens driving the deleterious immune responses in atherosclerosis is a promising new strategy. This can be achieved by inducing antigen-specific Tregs, which have the capacity to arrest pro-inflammatory responses and restore immune balance. In certain aspects, atherosclerosis resembles other inflammatory and autoimmune diseases like rheumatoid arthritis, multiple sclerosis or type 1 diabetes therefore the lessons learnt in clinical trials of immune modulating vaccines for these diseases can be used to advance in the development of similar strategies for atherosclerosis. Although there are still challenges to overcome in the clinical translation of pre-clinical results, immune-modulating vaccines for atherosclerosis can represent a leap forward in the treatment of CVD.

## 7 Outstanding questions

There are several questions that still need to be addressed in order to advance in the clinical development of tolerogenic vaccines for cardiovascular disease. To start, it is still not clear what aspects and how should we improve pre-clinical models to test the efficacy of this therapeutic approach and facilitate clinical translation.

Regarding the design of vaccine formulations for tolerance induction, the most optimal tolerogenic adjuvant for human use still needs to be defined.

On the other hand, there are still aspects of immune responses in atherosclerosis that need to be elucidated, for example, is there a single antigenic determinant driving immune response? The answer to this question will determine if we can develop a widely applicable tolerogenic vaccine or if we should follow a personalised strategy instead.

Related to the design of clinical trials, the preferred target population for a tolerogenic vaccine against atherosclerosis needs to be determined as well as the endpoints of these studies. Should this strategy be aimed only to patients with high-inflammatory risk or to the general population of atherosclerosis patients? Can we design large-scale clinical trials with endpoints that rely not only on general markers of cardiovascular disease but also on plaque morphology and immunophenotyping?

## 8 Search strategy and selection criteria

References for this review were identified using PubMed database. Only references related to clinical trials were included for the search terms “rheumatoid arthritis”, “multiple sclerosis”, “type 1 diabetes” in combination with “vaccine”, “tolerogenic vaccine”, “T regulatory cells”, “tolerogenic dendritic cells”. Other search terms used were “atherosclerosis”, “cardiovascular disease” in combination with “vaccination”, “tolerance”, “immune response”, “antigens”, “T regulatory cells”, “tolerogenic dendritic cells” “nanoparticles”, “adjuvants”. For this second part the search was not limited to clinical trials. Only articles published in English were included with preference for articles published in the past 10 years.

## Authors contribution

FLV, JK and BS designed the concept and outline of original draft. FLV wrote the original manuscript, JK and BS reviewed and supported the editing of the original manuscript. FLV, BS and JK edited final manuscript.

## Declaration of Competing Interest

The authors have nothing to disclose.
